# Effect of probiotic lozenges on improvement in tympanometric classification within 12 weeks in children aged 3–6 years with adenoid hypertrophy and otitis media with effusion undergoing non-surgical management: a randomized controlled trial

**DOI:** 10.3389/fcimb.2025.1736602

**Published:** 2026-02-09

**Authors:** Jun Shi, Yu Liu, Xiaoning Chen, Xiaofei Hou, He Chen, Luomeng Chen, Le Li

**Affiliations:** 1 Department of Otolaryngology, Affiliated Hospital of Nanjing University of Chinese Medicine, Nanjing, Jiangsu, China; 2 Department of Otolaryngology, Nanjing Integrated Traditional Chinese and Western Medicine Hospital Affiliated with Nanjing University of Chinese Medicine, Nanjing, Jiangsu, China

**Keywords:** adenoid hypertrophy, otitis media with effusion, placebo-controlled RCT, streptococcus salivarius K12, tympanogram classification

## Abstract

**Background:**

Otitis media with effusion (OME) is common among children aged 3–6 years with adenoid hypertrophy. Restoring middle-ear ventilation and clearing effusion are key goals during non-surgical management, and tympanogram classification serves as the main clinical indicator. The probiotic Streptococcus salivarius K12 (SSK12) can colonize the upper airway and inhibit pathogens, yet randomized trials using tympanogram classification as the primary endpoint are limited.

**Objective:**

This study aimed to evaluate the effect of daily SSK12 lozenges (≥1×10^9^ CFU) for 12 weeks on tympanogram classification improvement in children with adenoid hypertrophy and OME and to elucidate related microecological, pathogenetic, and audiological pathways, along with safety and internal validity measures.

**Methods:**

A single-center, randomized, double-blind, placebo-controlled RCT was conducted. All participants received standard non-surgical management and were allocated to either SSK12 or placebo lozenges for 12 weeks. The primary outcome was child-level tympanogram improvement (“B/C→A”) at week 12. Secondary analyses included ear-level results, tympanometric parameter changes, audiometric outcomes, and nasopharyngeal microbiota profiles. Logistic regression, generalized estimating equations (GEE), and linear mixed-effects models were employed to estimate adjusted odds ratios (aORs), interaction terms, and time effects.

**Results:**

Compared with placebo, SSK12 significantly increased the probability of tympanogram improvement (aOR=1.87, P = 0.003), with consistent benefits across subgroups (all p(interaction)>0.05). Ear-level analyses confirmed the advantage (all aOR>1, P<0.05), with moderate intra-child correlation (ρ≈0.33). SSK12 produced significant group × time interactions for tympanometric parameters—positive changes in ΔTPP and ΔYtm and decreased ΔTW (all P<0.01). Microbiological analyses demonstrated a marked increase in SSK12 colonization (both rate and load, P<0.001) and reduced loads of H. influenzae, S. pneumoniae, and M. catarrhalis (all P<0.01). Audiological outcomes improved, including greater decline in four-frequency pure-tone average (P = 0.002) and higher DPOAE conversion (aOR=2.06, P = 0.001). Incidences of upper respiratory infections and acute otitis media were reduced (both P<0.05). No significant adverse effects or blinding bias were observed.

**Conclusion:**

Daily SSK12 lozenges for 12 weeks significantly improved tympanogram classification and auditory function, mediated by enhanced probiotic colonization and pathogen suppression, with excellent safety and adherence. SSK12 represents a low-risk, biologically rational adjunct in the conservative management of pediatric OME associated with adenoid hypertrophy.

## 1 Introduction

In preschool children, otitis media with effusion often coexists with adenoid hypertrophy and eustachian tube dysfunction; recurrence or persistence can lead to conductive hearing loss and restricted speech development (Gunduz et al., 2025). In the non-surgical management phase, the core goals are to restore middle-ear ventilation and clear the effusion, and objective evaluation mainly relies on tympanogram classification and continuous parameters such as TPP, Ytm, and TW (Connolly et al., 2023; Shahnaz et al., 2023). Imbalance of nasopharyngeal microecology and colonization by pathogens are considered important upstream drivers (van den Broek et al., 2019); Streptococcus salivarius K12 is a resident upper airway bacterium with colonization and bacteriocin production, and biologically has the potential to improve middle-ear physiology through local microecological modulation (Al-Akel et al., 2024). Using lozenges to achieve sustained oropharyngeal exposure balances efficacy and accessibility, aligning with practical management needs during the observation period (Stasǩová et al., 2021). There remain key evidence gaps for this population. Conventional non-surgical interventions (e.g., nasal irrigation, autoinflation, intranasal steroids) show heterogeneous efficacy and have difficulty in reliably reversing middle-ear negative pressure in the short term (Nibhanupudy et al., 2024; Webster et al., 2023); previous probiotic studies mostly used upper respiratory infection or acute otitis media episodes as endpoints (Sarlin et al., 2023; Zhao et al., 2022), and randomized controlled trials using tympanogram classification as the primary endpoint while simultaneously quantifying microecological changes are lacking; previous studies rarely adopted ear-level correlation modeling, making it difficult to handle the structural correlation of bilateral ear outcomes (Sheng et al., 2022; Chen et al., 2022); colonization and pathogen load, tympanometric physiological indices, and audiological outcomes were often assessed separately, making it difficult to form integrated evidence for a causal chain. Clinically, a trial is still lacking that, within the context of standardized non-surgical management, centers on objective middle-ear physiological indicators and simultaneously addresses mechanism and safety to support the application of lozenge-based microecological intervention (Bourdillon and Edwards, 2021; Chen et al., 2020). This study conducted a single-center, randomized, double-blind, placebo-controlled trial to evaluate, in children aged 3–6 years with adenoid hypertrophy and otitis media with effusion undergoing non-surgical management, the effect of daily ≥1×10^9^ CFU Streptococcus salivarius K12 lozenges over 12 weeks on improvement in tympanogram classification, and to build an evidence chain linking ear physiology, microecology, and audiology using linear mixed-effects models and GEE, with systematic assessment of adherence, product viability, and safety. The trial showed that SSK12 achieved a higher probability of classification improvement than placebo, with time-dependent signals consistent with changes in TPP, Ytm, TW, audiological outcomes, and decreases in pathogen load; safety and acceptability were good, suggesting that introducing an oropharyngeal lozenge microecological intervention during the observation period has clinical translational value.

Our study focused on an objective and clinically meaningful primary endpoint, namely tympanogram improvement (Jerger type B/C→A) at week 12, in a well-defined pediatric OME population with adenoid hypertrophy (grade II–IV). Tympanogram normalization is a direct indicator of improved middle-ear ventilation and effusion resolution, making it an appropriate endpoint for assessing non-surgical interventions in this setting.

## 2 Materials and methods

### 2.1 Study design

This study was a single-center, randomized, double-blind, placebo-controlled trial. It was conducted in the Department of Otolaryngology-Head and Neck Surgery of our hospital and enrolled children aged 3–6 years with adenoid hypertrophy and otitis media with effusion (OME). All participants received standardized non-surgical management and were then randomly assigned to receive Streptococcus salivarius K12 (SSK12) lozenges or matched placebo lozenges for 12 consecutive weeks. The primary outcome was assessed at week 12. Study timeline: participant recruitment from March 1, 2020 to February 28, 2025; last case completed follow-up by May 31, 2025; database lock on June 30, 2025. The study was approved by the institutional ethics committee (approval No. XXXX), and written informed consent was signed by the legal guardians of all participants before enrollment.

### 2.2 Participants

Inclusion criteria: ① age ≥3 years and<7 years; ② at least one ear showed Jerger type B or C on tympanograms at both screening and baseline (14 ± 3 days apart); ③ medical history indicated that middle-ear effusion or negative middle-ear pressure had persisted for ≥6 weeks (Duration ≥6 weeks was determined by caregiver history; outpatient visit records were reviewed when available); ④ adenoid hypertrophy graded as II, III, or IV by nasopharyngeal endoscopy (graded by the percentage of the nasopharyngeal sagittal diameter occupied by the adenoid- e.g.: Grade II (25–50%), Grade III (50–75%), Grade IV (>75%); ⑤ the guardian signed written informed consent and was able to cooperate with follow-up. Exclusion criteria: ① prior adenoidectomy or tympanostomy tube insertion; ② use of any oral or nasopharyngeal probiotic preparation within 14 days before the trial; ③ systemic antibiotic therapy within 14 days before the trial; ④ confirmed immunodeficiency, congenital craniofacial malformations, cleft palate, or velopharyngeal insufficiency; ⑤ Children receiving systemic corticosteroids or other immunosuppressive medications within the preceding 3 months were excluded because these agents may influence immune responses, middle-ear inflammation, and probiotic colonization; ⑥ allergy to lozenge excipients (isomalt, xylitol, magnesium stearate, natural flavors) or to SSK12; ⑦ inability to take the lozenge as required (failed the swallowing risk assessment); (8) other conditions judged by the investigators to affect adherence or safety. (9) Children with a recent tympanic membrane perforation or a history of tympanic membrane surgery were excluded to avoid confounding tympanometric measurements and hearing outcomes, as membrane integrity directly affects middle-ear mechanics. (10) Children with severe hearing loss, defined as a four-frequency pure-tone average (0.5, 1, 2, and 4 kHz) >40 dB HL in either ear, were excluded because such loss is unlikely to be attributable solely to otitis media with effusion and may indicate alternative or irreversible pathology. (11) Children with chronic suppurative otitis media, including persistent otorrhea or active middle-ear infection, were excluded because the study targeted non-suppurative OME, and inclusion of CSOM would introduce heterogeneity in pathophysiology and treatment response. (12) Children with a known allergy or hypersensitivity to *Streptococcus salivarius* K12 or any lozenge excipients were excluded for safety reasons and to minimize the risk of treatment-related adverse events. This confirmation interval was chosen to minimize enrollment of transient OME and to strengthen persistence criteria.

#### 2.2.1 Recruitment process and baseline assessment

Children meeting the age criteria were preliminarily screened in the outpatient clinic. After completion of informed consent, screening assessments were performed (Day -14 to Day -1): medical history and physical examination, nasopharyngeal endoscopic adenoid grading, first tympanogram and audiological assessment, and collection of saliva and nasopharyngeal swab samples. At the baseline visit (Day 0), the tympanogram was repeated to confirm the classification and randomization was completed. Baseline records included demographic information, disease burden (unilateral/bilateral, duration of illness), passive smoking exposure, daycare/kindergarten attendance, and concomitant treatments in the past 8 weeks.

### 2.3 Sample size estimation, randomization, and blinding

The primary outcome was the child-level classification improvement rate at week 12. Assuming an improvement rate of 50% in the trial group and 35% in the control group (a difference of 15 percentage points), two-sided α=0.05, and power of 80%. According to the sample size formula for comparing two independent proportions (Kim, 2016):

(1) $$\text{n}={\left(\frac{{\text{Z}}_{1-\alpha /2}\sqrt{2\bar{p}(1-\bar{p})}+{\text{Z}}_{1-\beta }\sqrt{{\text{p}}_{1}(1-{\text{p}}_{1})+{\text{p}}_{2}(1-{\text{p}}_{2})}}{{\text{p}}_{1}-{\text{p}}_{2}}\right)}^{2},\text{where }\bar{p}=\frac{{\text{p}}_{1}+{\text{p}}_{2}}{2}$$

Substituting the values yields 170 participants per group in Equation 1. Considering 10% loss to follow-up and non-adherence, we planned to enroll 190 per group, with a total sample size of 380. A 1:1 allocation ratio was used. An independent statistician generated variable block-length computer randomization sequences (block sizes 4 and 6), with stratification factors including baseline classification of the main affected ear (Jerger type B or C) and whether bilateral involvement was present. Allocation concealment was implemented via a centralized interactive web-based system, with randomization codes held by the pharmacy department. The placebo lozenges were identical in appearance, taste, texture, and packaging to the SSK12 lozenges and contained the same excipients without viable bacteria. Study participants, caregivers, investigators, and outcome assessors remained blinded to treatment allocation throughout the trial. At week 12, blinding success was evaluated (participants and assessors guessed group assignment and recorded the basis).

### 2.4 Interventions and control

#### 2.4.1 Streptococcus salivarius K12 lozenge regimen

The trial group used SSK12 lozenges (BLIS Technologies Limited, Dunedin) (each lozenge contained freeze-dried SSK12 viable bacteria ≥1×10^9^ CFU, with excipients of isomalt, xylitol, magnesium stearate, and natural flavors). One lozenge was taken nightly before bedtime and allowed to dissolve completely, with a sucking duration of no less than 5 minutes, and no food or drink within 30 minutes after administration (Poorni et al., 2022). The SSK12 lozenges were stored at 2–8 °C, protected from heat and moisture, in accordance with manufacturer specifications. Lot numbers were recorded for all dispensed products to monitor lot-to-lot consistency. Product viability, expressed as colony-forming units (CFU), was verified at dispensing and at predefined intervals during the study using [specify method, e.g., culture-based CFU enumeration or manufacturer-validated assay], confirming that the labeled dose (≥1 × 10^9^ CFU per lozenge) was maintained throughout the study period. Adherence was assessed by parents’ daily dosing records and cross-checked with counts of dispensed/returned lozenges, and adherence was defined as actual intake ≥80% of the planned dose.

#### 2.4.2 Control measures and management of concomitant treatments

The control group used placebo lozenges identical to the trial group in appearance, odor, taste, and packaging, without viable bacteria, with the same dosing regimen and adherence assessment. Both groups received standardized non-surgical management: health education, avoidance of secondhand smoke exposure, and isotonic saline nasal spray twice daily. During the trial, initiating new oral or oropharyngeal/nasopharyngeal probiotics was prohibited, as was self-provided autoinflation training devices and the use of intranasal or oral corticosteroids. In the event of acute otitis media, rescue antibiotic therapy could be initiated per prespecified diagnostic criteria, as determined by the study physician, with complete recording of drug name, dose, and course.

#### 2.4.3 Standard non-surgical management

Standard non-surgical management was protocolized and applied equally to both groups. This included caregiver education regarding the natural history of otitis media with effusion, avoidance of environmental risk factors (e.g., second-hand smoke exposure), and supportive nasal care with isotonic saline as needed. No routine ENT procedures, autoinflation devices, intranasal or systemic corticosteroids, decongestants, antihistamines, or additional probiotics were permitted during follow-up.

#### 2.4.4 Antibiotic therapy

Antibiotic therapy was not used prophylactically and was permitted only as rescue treatment for physician-diagnosed acute otitis media based on prespecified clinical criteria. All antibiotic use (agent, dose, duration, and timing) was prospectively recorded.

#### 2.4.5 Control of confounders in microbiological analyses

Variables known to influence nasopharyngeal microbiota were prospectively recorded, including recent upper respiratory infection, systemic antibiotic exposure within the preceding [e.g., 4–8] weeks, and day-care/school attendance. These factors were included as covariates in multivariable regression models assessing SSK12 colonization and otopathogen loads. Sensitivity analyses excluding participants with recent antibiotic exposure were also performed to evaluate the robustness of microbiological findings.

### 2.5 Outcome measures

#### 2.5.1 Primary outcome: improvement in tympanogram classification

The primary outcome was child-level classification improvement: Tympanogram improvement at week 12 was defined as conversion to Jerger type A in ≥1 ear that was classified as Jerger type B or C at baseline. Participants were eligible only if they had at least one ear with Jerger type B or C at both screening and baseline (Day 0). For children with unilateral OME, improvement was assessed in the affected ear. For children with bilateral OME, the child was classified as improved if either ear converted to type A at week 12; if one ear improved and the other did not, the child was still classified as improved. Ears with baseline type A were excluded from improvement assessment. Jerger classification criteria were as follows (Othman et al., 2024): type A, a single distinct peak is present, the tympanometric peak pressure (TPP) is between −100 and +50 daPa, and the static admittance (Ytm) is ≥0.2 mL; type C, a single peak is present and TPP<−100 daPa, with Ytm ≥0.2 mL; type B, the curve has no identifiable peak or is plateau-shaped, Ytm<0.2 mL, and the ear canal volume (ECV) is within the age-appropriate range. The baseline classification was determined by the average of two measurements; at week 12, the average of two repeated measurements was used.

#### 2.5.2 Primary endpoint (child-level): Secondary outcomes

① ear-level classification improvement rate; ② changes from baseline in TPP, Ytm, tympanometric width (TW), and ECV; ③ changes in four-frequency pure-tone average (PTA) at 0.5/1/2/4 kHz and the proportion of distortion product otoacoustic emission DPOAE conversion (DPOAE conversion was defined as a change from absent (fail) to present (pass) responses between baseline and follow-up assessments. Presence was determined using standard signal-to-noise ratio criteria across the tested frequencies) from negative to positive (Garbaruk et al., 2021); ④ detection rate and changes in load of SSK12 colonization in saliva and nasopharynx; ⑤ detection rates and changes in loads of nasopharyngeal pathogens (Haemophilus influenzae, Streptococcus pneumoniae, Moraxella catarrhalis); ⑥ numbers of acute upper respiratory infection episodes, acute otitis media episodes, and systemic antibiotic use within the 12-week follow-up; ⑦ incidence of rescue surgical intervention (tympanostomy tube insertion or adenoidectomy).

#### 2.5.3 Multiple comparisons

The study was powered for the primary endpoint. Secondary outcomes were prespecified and analyzed as supportive. To address multiplicity across secondary endpoints (audiological, microbiological, URI/AOM outcomes), we applied the Benjamini–Hochberg false discovery rate (FDR) procedure to secondary-outcome p-values. Both unadjusted and FDR-adjusted p-values are reported; interpretation emphasizes the primary endpoint.

#### 2.5.4 Safety and adverse events

All adverse events and serious adverse events were recorded, including time of onset, duration, severity, management, outcome, and relationship to the intervention. Key monitoring included oral mucosal discomfort, signs of oral candidiasis, abdominal pain, nausea, vomiting, diarrhea, rash, and fever. In the event of a serious adverse event or when the investigator judged that there was an important risk, the study drug was immediately discontinued and necessary medical management was implemented.

### 2.6 Assessments and measurements

#### 2.6.1 Tympanometric measurements

A 226 Hz probe tone at 85 dB SPL was used, with a pressure range of +200 to −400 daPa and a pump speed of 50 daPa/s. The device was an Interacoustics AT235 tympanometer (same model and same software version throughout), calibrated once monthly. Ambient noise during measurement was<50 dB A. Participants were seated, and an earplug fitting the external auditory canal was selected to ensure airtightness. Each ear underwent 2 independent measurements, and the mean was taken as the valid result for that visit; if the difference between the two exceeded the prespecified threshold (difference in TPP >30 daPa or difference in Ytm >0.1 mL) (Merchant et al., 2021), an additional measurement was performed and the median of the 3 measurements was used. Visit time points: baseline, week 4, week 8, week 12. Original curve files were uploaded to a central reading platform and were read in a blinded manner by 2 trained audiologists, with discrepancies adjudicated by a third senior audiologist.

#### 2.6.2 Microecology and colonization/pathogen load assessment

At baseline, week 4, and week 12, two milliliters of unstimulated saliva were collected and one nasopharyngeal flocked swab was obtained per participant; swabs were placed into tubes containing 1 mL of preservation solution and stored at −80 °C within 30 minutes after sampling. DNA was extracted using the QIAamp DNA Mini Kit, strictly following the manufacturer’s instructions, with negative extraction controls and positive controls set for each run. Microecological indices were measured by real-time quantitative PCR (qPCR): for SSK12 colonization, the 16S rRNA gene of Streptococcus salivarius was selected as the target, combined with detection of salivaricin-related genes (salA and salB), and quantification was performed using the standard-curve method, expressed as copies/μL; for pathogens (H. influenzae, S. pneumoniae, and M. catarrhalis), specific primers and probes were designed and quantified separately, with results also quantified by the standard-curve method. Amplification was performed on an Applied Biosystems 7500 Fast instrument. Each batch included negative and positive controls, and the testing personnel remained blinded to participants’ group assignments.

#### 2.6.3 Audiological and event outcome assessment

Audiology: visual reinforcement audiometry (VRA) was used for ages 3–4 years, and conditioned play audiometry (CPA) for ages 5–6 years, calculating the four-frequency pure-tone average (air-conduction thresholds at 0.5/1/2/4 kHz, dB HL). Contralateral masking was applied when necessary. DPOAE at 2, 3, and 4 kHz recorded “present/absent” (Robler et al., 2023).

Event outcomes: episodes of acute upper respiratory infection and acute otitis media were collected by both parental paper diaries and weekly telephone follow-up, and confirmed by the study physician according to prespecified clinical and otoscopic criteria; all systemic antibiotic prescriptions were recorded, including drug name, dose, and course.

#### 2.6.4 Follow-up and adherence

Visit and follow-up schedule: baseline (Day 0) completed randomization and first dispensing; outpatient follow-up at week 4 and week 8 (collection of empty blister packs, verification of adherence, assessment of adverse events, repeated tympanometry and sampling); final visit at week 12 (assessment of the primary outcome and all secondary outcomes and safety). Weekly telephone follow-up was conducted to verify medication use and events. Adherence<80% was defined as poor adherence and was included in sensitivity analyses. Tympanometry was performed using standardized equipment and protocols with a 226-Hz probe tone, pressure sweep from +200 to −400 daPa, and a constant pump speed. Tympanometric parameters—including tympanometric peak pressure (TPP), static admittance (Ytm), and tympanometric width (TW)—were extracted from digitally stored curves. Adherence to the study intervention was monitored using caregiver-maintained daily dosing diaries and counts of dispensed and returned lozenges/blister packs at each scheduled visit. Weekly telephone contacts were used to reinforce adherence and document missed doses. Adherence was calculated as the proportion of prescribed doses consumed, and ≥80% adherence was defined *a priori* as protocol compliant.

### 2.7 Data management and quality control

Data were entered into an electronic case report form (eCRF, based on REDCap) with built-in logic checks and missing-value alerts; study data were double-checked, and the audit trail was automatically recorded. Original tympanogram data and reading results were fully retained. Devices were calibrated monthly with records kept, and study personnel completed standardized training and assessment prior to enrollment. Microecology specimens were maintained under cold chain throughout with chain-of-custody records. Before database lock, 100% of critical variables were verified and a 10% source data audit was performed.

### 2.8 Statistical analysis

The primary analysis used the intention-to-treat set (ITT), including all randomized participants who took at least one dose of the study preparation; the per-protocol set (PP) was used for sensitivity analyses. The safety set included all participants who took at least one dose of the study drug and completed safety evaluation. The main unit of analysis was the child level; ear-level indices used correlation methods to handle within-child correlation between ears.

The primary binary outcome was analyzed using multivariable regression models, and results are reported as adjusted odds ratios (aORs) with 95% confidence intervals and p-values. Longitudinal changes in continuous tympanometric parameters (tympanometric peak pressure [TPP], static admittance [Ytm], and tympanometric width [TW]) were analyzed using linear mixed-effects models including fixed effects for treatment group, time, and treatment-by-time interaction, with participant-level random intercepts. The study was analyzed by multivariable logistic regression adjusting for covariates including age (months), baseline classification of the main affected ear (type B or C), bilateral involvement, and enrollment quarter. Analyses were conducted at both the child level and ear level to appropriately address the bilateral structure of the data. The primary endpoint was analyzed at the child level, consistent with clinical decision-making. For ear-level binary outcomes, generalized estimating equations (GEE) with an exchangeable working correlation were used to account for within-child correlation between ears. Longitudinal changes in continuous tympanometric parameters were analyzed using linear mixed-effects models with participant-level random intercepts. Ear-level improvement in tympanogram classification was analyzed using a generalized estimating equations (GEE) binomial logit model with an exchangeable working correlation structure; changes in continuous tympanometric parameters and hearing thresholds were analyzed using linear mixed-effects models, with participants as random intercepts and time points, group, and their interaction as fixed effects. For microecological outcomes, colonization status (present/absent) was analyzed with a GEE binomial logit model, and load data, after logarithmic transformation, were analyzed with linear mixed-effects models. The working correlation (ρ) was estimated from GEE and reported descriptively; no ICC adjustment was applied to powering because the primary endpoint was child-level. Count-type event outcomes (numbers of upper respiratory infection and acute otitis media episodes) were analyzed using negative binomial regression with follow-up time as the offset. Data conforming to a normal distribution were presented as mean ± standard deviation, those not conforming to normality as median and interquartile range, and count data as number and percentage. Between-group comparisons for skewed continuous variables used the Mann–Whitney U test, the 95% confidence interval for the difference between two proportions was calculated using the Newcombe method, and significance testing used the χ² test or Fisher’s exact test according to expected counts.

Missing data for the primary outcome were handled by multiple imputation (chained equations, m=20), with the imputation model including treatment group, baseline classification, bilateral involvement, age, and enrollment quarter; sensitivity analyses included a conservative analysis treating “missing as not improved” and blinded multiple imputation. Subgroup analyses were pre-specified *a priori* to explore consistency of treatment effects across clinically relevant strata. These included:

- Baseline tympanogram type (Jerger type B vs. type C)
- Laterality of disease (unilateral vs. bilateral OME)
- Adenoid hypertrophy grade (II, III, IV)
- Age group (e.g.,<6 years vs. ≥6 years)
- Season of enrollment (spring–summer vs. autumn–winter)

Pre-specified analyses: The primary outcome (week-12 tympanogram normalization, B/C→A) and the prespecified secondary outcomes (tympanometric parameters [TPP, Ytm, TW], DPOAE status, four-frequency PTA, targeted pathogen loads/SSK12 detection, rescue antibiotic use, and rescue surgery) were defined *a priori* in the study protocol/statistical analysis plan before unblinding and database lock. The primary endpoint was analyzed as the main confirmatory test; secondary outcomes were considered supportive.

Pre-specified subgroup analyses: Subgroup analyses were pre-specified for age group, baseline tympanogram type (B vs C), unilateral vs bilateral disease, adenoid hypertrophy grade (II–IV), and season of enrollment. Subgroup effects were assessed using interaction terms and interpreted cautiously.

Exploratory analyses: Any additional analyses not explicitly listed above (e.g., alternative outcome definitions beyond the primary endpoint, correlations between audiological and tympanometric changes, and additional stratified correlation estimates) were conducted as exploratory analyses and are reported as hypothesis-generating without formal multiplicity correction.

Handling of missing data: The primary analysis was conducted according to the intention-to-treat (ITT) principle, including all randomized participants who received at least one dose of the study product. Missing outcome data were addressed using multiple imputation by chained equations, assuming data were missing at random. Imputation models included treatment group, baseline tympanogram type, age, adenoid grade, and available outcome measures. A per-protocol (PP) analysis, including participants with ≥80% adherence and complete primary outcome data, was performed as a sensitivity analysis to assess robustness.

## 3 Results

### 3.1 Participant enrollment and baseline characteristics

**Figure 1:**
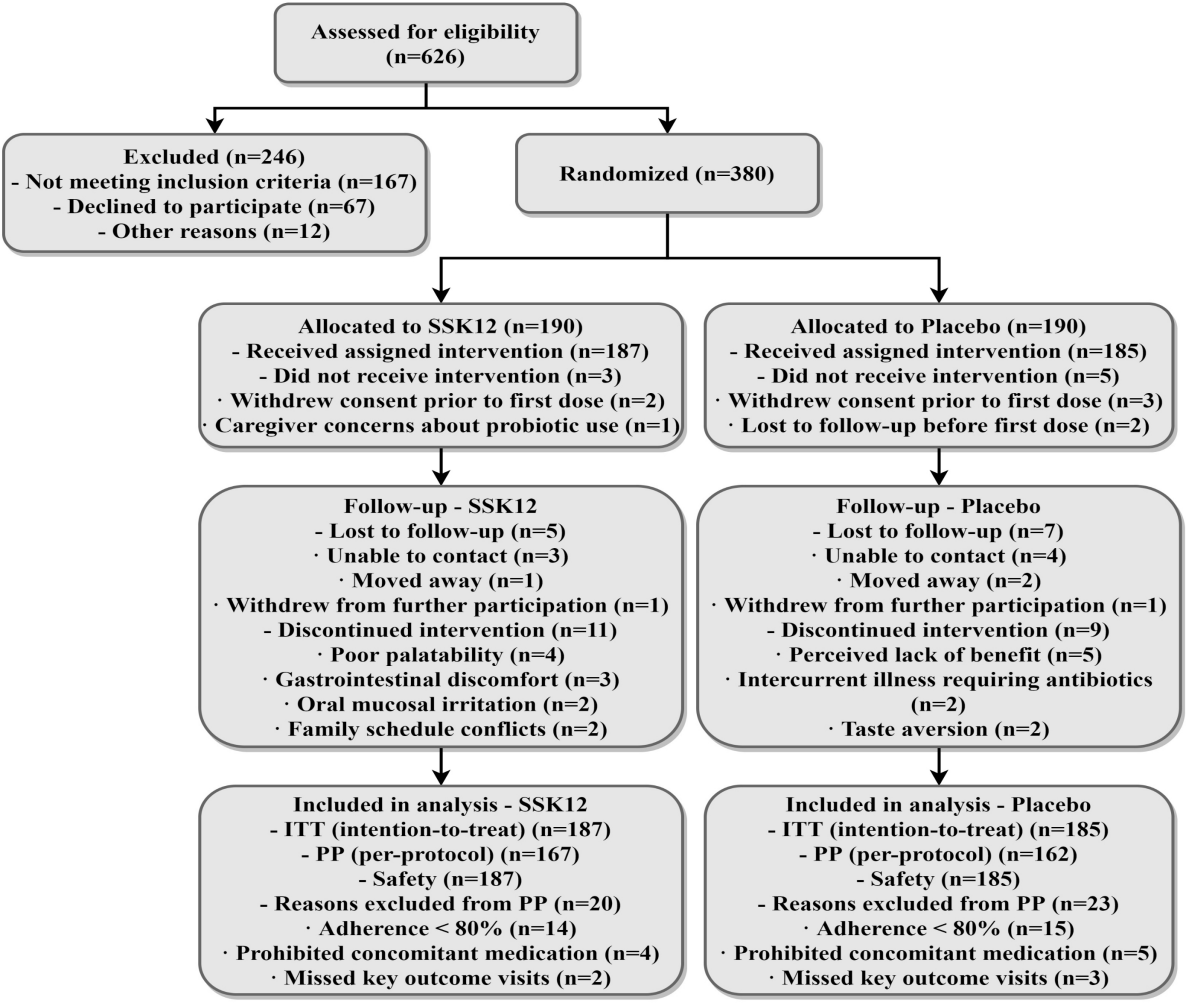
CONSORT flow diagram of participant recruitment, randomization, follow-up, and analysis.

A total of 626 children were screened, of whom 380 met the inclusion criteria and were randomized; the numbers who actually started the intervention were 187 in the SSK12 group and 185 in the placebo group (190 per group randomized), and 329 completed the intervention and entered the per-protocol analysis (PP set) (**Figure 1**). The two groups were balanced in age, sex, seasonal distribution, daycare/kindergarten attendance, and passive smoking proportion (|SMD| all<0.10). There were no obvious between-group differences in duration of illness, side of involvement, proportion of recurrent cases, adenoid grading, baseline hearing loss, or tympanometric characteristics (|SMD| all<0.10) (**Table 1**).

**Table 1:** Baseline characteristics of the study population.

Characteristic	SSK12 Group (n = 115)	Placebo Group (n = 115)	*p* value
Demographics			
Age, years (mean ± SD)	5.8 ± 1.6	5.7 ± 1.5	0.72
Age category, n (%)			
<6 years	62 (53.9)	65 (56.5)	0.69
≥6 years	53 (46.1)	50 (43.5)	
Male sex, n (%)	67 (58.3)	64 (55.7)	0.68
Clinical characteristics			
Duration of OME, weeks (median [IQR])	9.0 [7.0–12.0]	9.0 [7.0–11.5]	0.81
Laterality of OME, n (%)			
Unilateral	41 (35.7)	39 (33.9)	0.77
Bilateral	74 (64.3)	76 (66.1)	
Baseline tympanogram type, n (%)			
Type B	69 (60.0)	71 (61.7)	0.79
Type C	46 (40.0)	44 (38.3)	
Adenoid hypertrophy grade, n (%)			
Grade II	29 (25.2)	31 (27.0)	0.88
Grade III	54 (47.0)	52 (45.2)	
Grade IV	32 (27.8)	32 (27.8)	
Baseline PTA (dB HL), mean ± SD	28.4 ± 6.3	28.7 ± 6.1	0.71
Baseline DPOAE present, n (%)	18 (15.7)	16 (13.9)	0.69
Other factors			
≥2 AOM episodes in past 6 months, n (%)	37 (32.2)	35 (30.4)	0.77
Passive smoking exposure, n (%)	28 (24.3)	30 (26.1)	0.75
Season of enrollment, n (%)			
Spring–Summer	56 (48.7)	54 (47.0)	0.80
Autumn–Winter	59 (51.3)	61 (53.0)	

### 3.2 Intervention exposure, adherence, and trial execution quality

The Mann–Whitney U test and χ² test were used. There were no significant differences between the two groups in adherence ≥80%, medication interruption ≥3 days, violations of prohibited concomitant treatments, or initiation of rescue antibiotic therapy (all P>0.05); the SSK12 group had slightly higher actual doses taken and percentage adherence (both P<0.05), but with small magnitudes (**Table 2**). By spot checks, viable counts of SSK12 lozenges were stably above the labeled amount both before dispensing and at week 6, with no significant attenuation; viable counts in the placebo group were not detected throughout; storage temperatures of both preparations met the prespecified cold-chain requirements during the study (**Table 3**). Blinding success was evaluated using the Bang blinding index. Judgments of group assignment by participants and assessors were relatively balanced (BI values in each group were close to 0, all P>0.05), indicating good implementation of blinding with no obvious identification tendency (**Table 4**). Consistency of Jerger classification readings on tympanograms was high (Cohen κ=0.88), and repeatability of TPP, Ytm, and TW measurements was good to excellent (all ICC≥0.89), indicating stable and reliable measurement methods (**Table 5**).

**Table 2:** Intervention exposure and adherence overview.

Indicator	SSK12 group (n=187)	Placebo group (n=185)	H–L or RD (95% CI)	p
Planned doses (lozenges)	84	84	—	—
Actual doses taken (lozenges)	80 [76–83]	79 [74–82]	H–L: 1.00 (0.003~2.002)	0.041
Adherence ≥80%	173 (92.51%)	170 (91.89%)	RD: 0.62% (−7.20%~8.43%)	0.823
Adherence (%)	94.13 ± 8.72	92.41 ± 9.35	H–L: 1.70% (0.40%~3.00%)	0.012
Medication interruption ≥3 days	21 (11.23%)	19 (10.27%)	RD: 0.96% (−8.02%~9.88%)	0.765
Violation of prohibited concomitant treatments	4 (2.14%)	5 (2.70%)	RD: −0.56% (−5.33%~4.21%)	0.750
Initiation of rescue antibiotic therapy	21 (11.23%)	28 (15.14%)	RD: −3.91% (−13.54%~5.87%)	0.265

**Table 3:** Viable counts and quality control of study preparations.

Type	Spot-check time point	Viable count per lozenge (CFU/lozenge)	log₁₀(CFU/lozenge)	Deviation from labeled amount (%)	Storage temperature monitoring (°C)
SSK12	Before dispensing	1.24×10^9^ ± 0.11×10^9^	9.09 ± 0.04	24.37	2.72–7.46
	Week 6 of administration	1.17×10^9^ ± 0.10×10^9^	9.07 ± 0.05	17.29	2.87–7.58
Placebo	Before dispensing	Not detected	—	—	2.65–7.19
	Week 6 of administration	Not detected	—	—	2.81–7.52

Viable counts were determined by the plate count method.

**Table 4:** Blinding success evaluation.

Group	Guessed SSK12	Guessed placebo	Uncertain	BI (95% CI)	p value
Participants—SSK12 group	62 (34.07%)	57 (31.32%)	63 (34.62%)	0.03 (−0.09 ~ 0.14)	0.647
Participants—placebo group	59 (33.15%)	57 (32.02%)	62 (34.83%)	−0.01 (−0.13 ~ 0.11)	0.853
Assessors—SSK12 group	53 (29.12%)	51 (28.02%)	78 (42.86%)	0.01 (−0.10 ~ 0.12)	0.845
Assessors—placebo group	56 (31.46%)	55 (30.90%)	67 (37.64%)	−0.01 (−0.12 ~ 0.11)	0.924

BI close to 0 indicates good blinding; values approaching 1 or −1 indicate a significant tendency toward correct (or systematically incorrect) guesses.

**Table 5:** Consistency of central reading for tympanograms and measurement repeatability.

Indicator	Sample size (n ears)	Statistic (95% CI)
Consistency of Jerger classification by two readers	372	Cohen κ = 0.88 (0.84~0.91)
Repeatability of TPP (type C ears)	166	ICC(2,1) = 0.94 (0.92~0.96)
Repeatability of Ytm	372	ICC(2,1) = 0.92 (0.90~0.94)
Repeatability of TW (type C ears)	166	ICC(2,1) = 0.89 (0.86~0.92)

### 3.3 Primary outcome: improvement in tympanogram classification at week 12 (child level)

**Figure 2:**
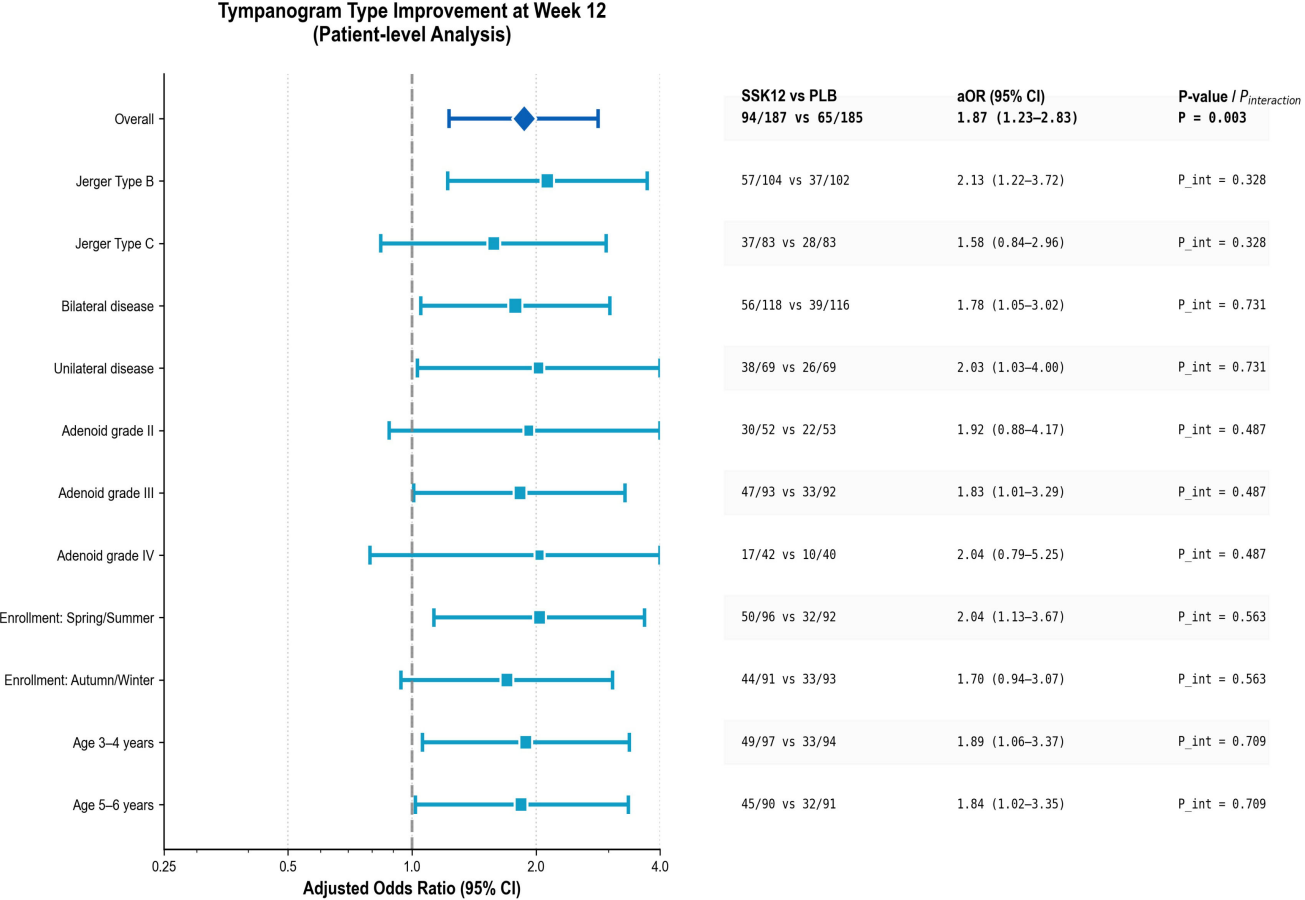
Forest plot of effects on the primary outcome at week 12.

By multivariable logistic regression, SSK12 had a higher classification improvement rate than placebo at week 12 (aOR=1.87, P = 0.003). Across prespecified subgroups (Jerger Type B/C, bilateral involvement, adenoid grade, enrollment quarter, age strata), the direction of effect was consistent. In models containing baseline covariates, “treatment × subgroup” interaction terms were added and tested separately, with no significant effect modification observed (all p_interaction>0.05) (**Figure 2**).

At week 12, tympanogram normalization (B/C→A) occurred in 50.4% (58/115) of children in the SSK12 group compared with 35.7% (41/115) in the placebo group, corresponding to an absolute risk difference of 14.7 percentage points. This yields a number needed to treat (NNT) of 7. In adjusted analysis, this difference corresponded to an adjusted odds ratio (aOR) of 1.87.

### 3.4 Tympanogram-related secondary outcomes

**Figure 3:**
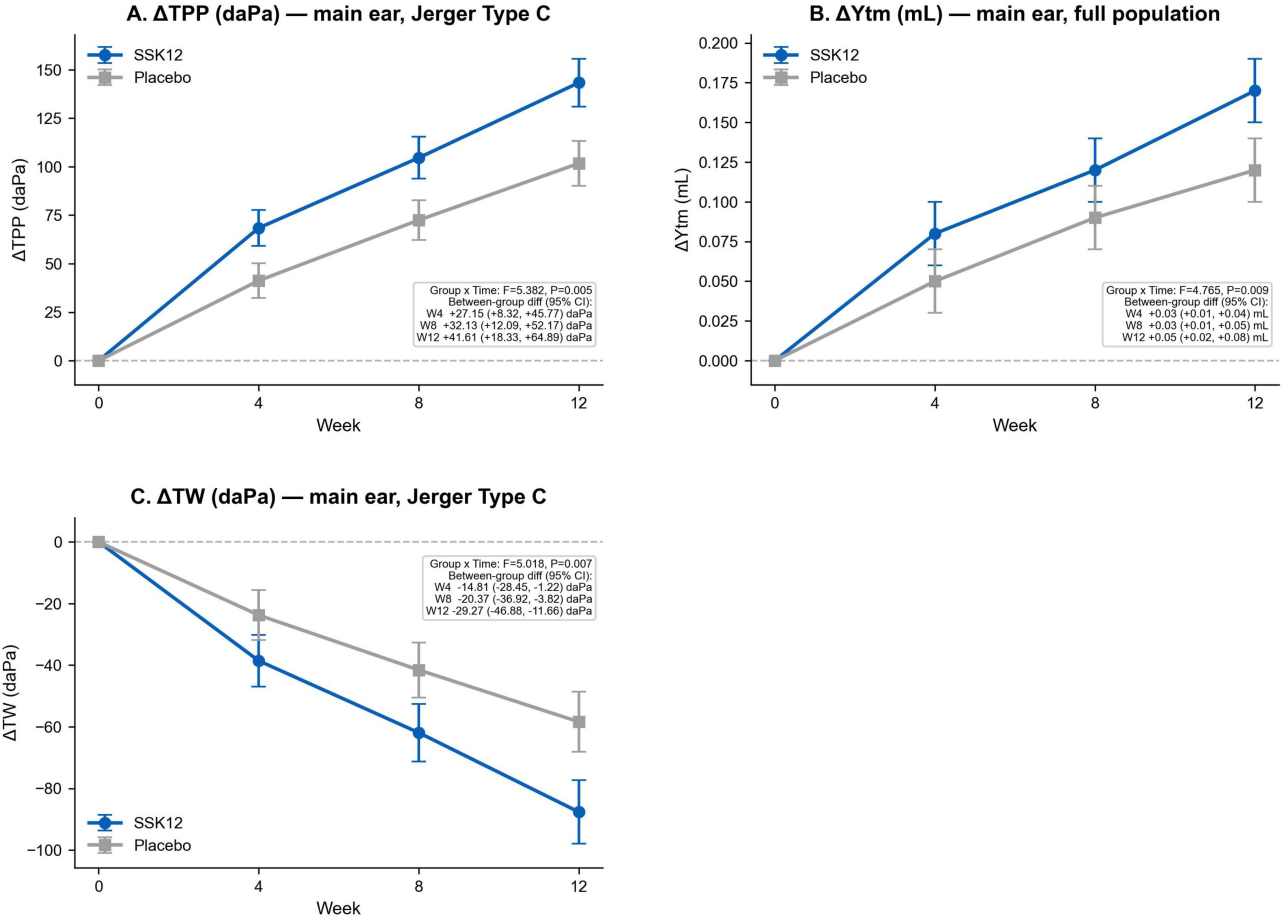
Longitudinal changes in continuous tympanogram parameters. **(A)** ΔTPP; **(B)** ΔYtm; **(C)** ΔTW.

A linear mixed-effects model was used to analyze changes from baseline in tympanogram parameters (Δ, value at each follow-up minus baseline). Compared with the placebo group, the SSK12 group showed greater magnitudes of increase in ΔTPP and ΔYtm and decrease in ΔTW, and the between-group differences increased with longer follow-up; all “group × time” interaction effects were statistically significant (all P<0.01) (**Figure 3A, B, C**). At the ear level, a GEE binomial logit model (exchangeable working correlation structure) was used to evaluate classification improvement of “B/C→A.” The results showed that the probabilities of improvement were higher in the SSK12 group than in the placebo group both overall and in ears with Jerger types B/C (all aOR>1, all P<0.05); the model-estimated working correlation coefficient was ρ≈0.33, indicating moderate correlation between outcomes of the two ears within the same child (**Table 6**).

**Table 6:** Ear-level classification improvement rate (week 12) and GEE model estimates.

Population/ ear type	SSK12 group	Placebo group	aOR_GEE (95% CI)	p value	ρ (working correlation coefficient)
Overall ear level	125/292 (42.74%)	81/286 (28.39%)	1.86 (1.31~2.66)	0.001	0.33
Jerger Type B	76/160 (47.50%)	51/158 (32.28%)	1.90 (1.20~3.01)	0.006	0.32
Jerger Type C	49/132 (37.12%)	30/128 (23.44%)	1.93 (1.10~3.37)	0.022	0.35

Analysis based on complete cases (participants with missing ear-level classification at week 12 were not included in this table).

### 3.5 Microecological and pathogenetic results

**Figure 4:**
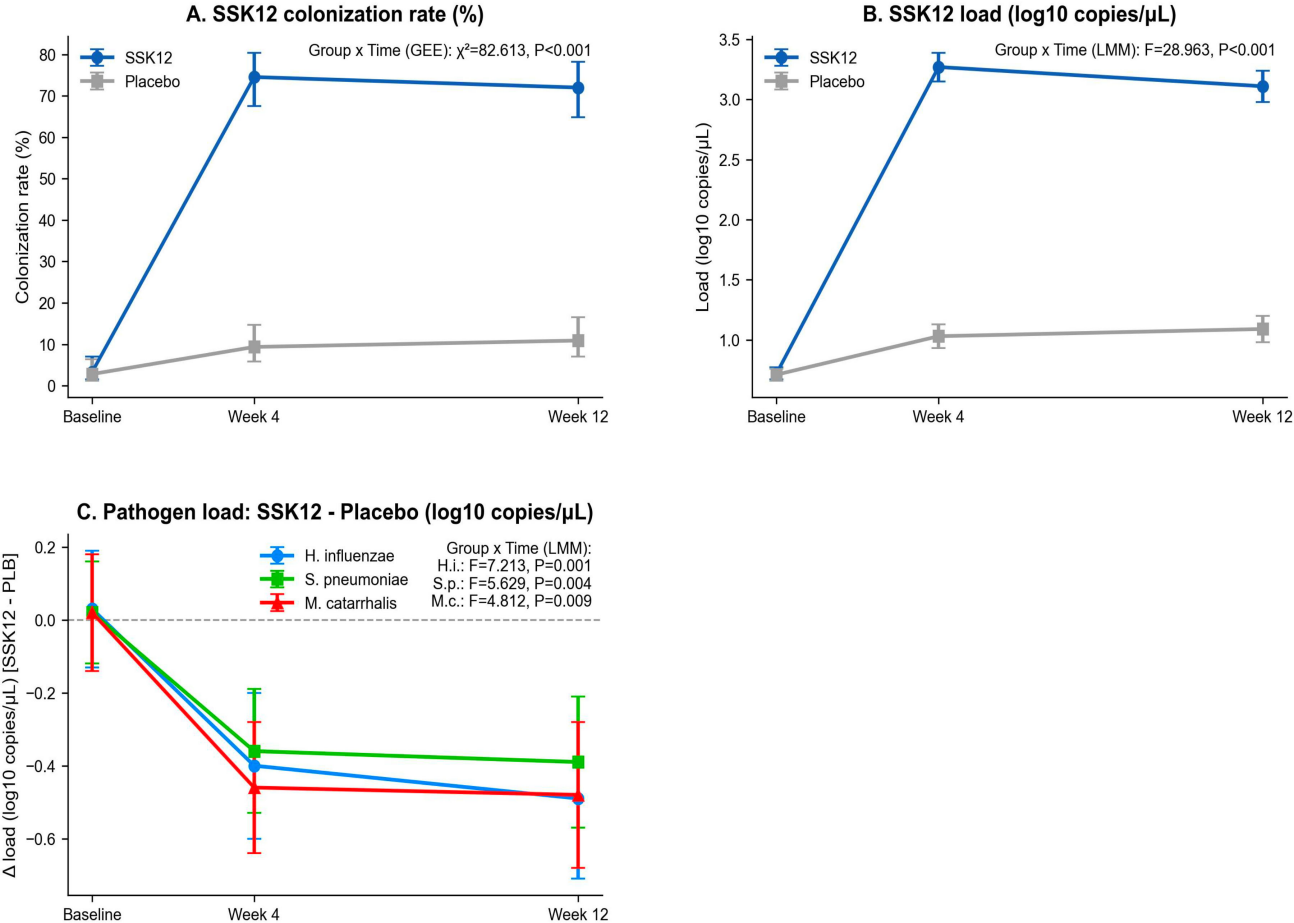
Microecological outcomes. **(A)** SSK12 colonization rate. **(B)** SSK12 load. **(C)** Pathogen load (SSK12 − Placebo). qPCR used standard-curve quantification; results below the limit of quantification were set to half the limit and then log-transformed.

GEE (binomial logit) and linear mixed-effects models were used to assess microecological outcomes. Compared with placebo, the SSK12 group showed more pronounced increases over time in colonization rate and load, with significant group × time interactions (both P<0.001); loads of the three pathogens (H. influenzae, S. pneumoniae, M. catarrhalis) declined more markedly over time, with significant group × time interactions as well (all P<0.01) (**Figure 4A, B, C**).

### 3.6 Audiological and otologic functional outcomes

**Figure 5:**
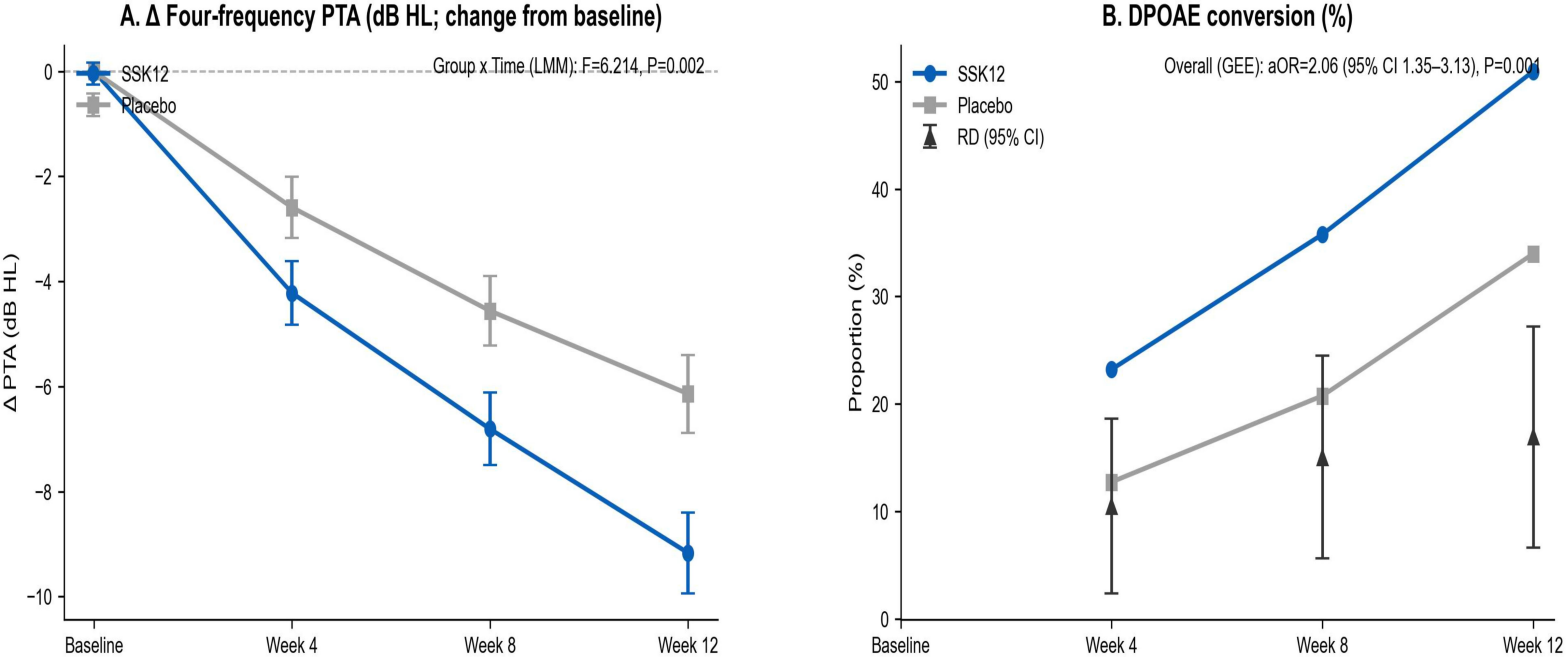
Audiological and otologic functional outcomes. **(A)** Δ Four-frequency PTA. **(B)** DPOAE conversion.

Linear mixed-effects model and GEE (binomial logit) analyses showed that the SSK12 group had a larger decrease in Δ four-frequency pure-tone average (group × time interaction P = 0.002), and a higher overall probability of DPOAE conversion from negative to positive (aOR=2.06, P = 0.001), suggesting a beneficial impact on hearing and otologic function (**Figure 5A, B**).

### 3.7 Event outcomes

Negative binomial regression (log link, offset=person-weeks) was used to analyze event outcomes. Compared with placebo, the SSK12 group had lower incidences of upper respiratory infection and acute otitis media (both aIRR<1, both P<0.05); between-group differences in the incidences of systemic antibiotic use and rescue surgical intervention were not statistically significant (both P>0.05) (**Table 7**).

**Table 7:** Event outcomes.

Outcome event	events/person-weeks; per 100 person-weeks		aIRR	95% CI	p	Overdispersion parameter θ
	SSK12 group	Placebo group				
Upper respiratory infection episodes	153 / 2,142.73 (7.14)	189 / 2,118.35 (8.92)	0.79	0.65~0.96	0.017	1.46
Acute otitis media episodes	38 / 2,142.73 (1.77)	55 / 2,118.35 (2.60)	0.69	0.50~0.95	0.021	1.12
Systemic antibiotic prescriptions	27 / 2,142.73 (1.26)	36 / 2,118.35 (1.70)	0.74	0.49~1.12	0.157	0.98
Rescue surgical intervention	2 / 2,142.73 (0.09)	4 / 2,118.35 (0.19)	0.54	0.10~2.83	0.487	0.79

Person-weeks were calculated as actual follow-up days/7; rescue surgical intervention was counted once by occurrence (yes/no).

### 3.8 Safety and adverse events

Safety outcomes were analyzed using Fisher’s exact test. The results showed no statistically significant differences between groups in the incidence of adverse events, serious adverse events, or discontinuation due to adverse events (all P>0.05), indicating that the overall safety of SSK12 was similar to that of placebo (**Table 8**).

**Table 8:** Adverse events by treatment group (Safety population).

Adverse Event	SSK12 (n = 115)	Placebo (n = 115)
Any adverse event, n (%)	24 (20.9)	26 (22.6)
Severity		
Mild	18 (15.7)	20 (17.4)
Moderate	6 (5.2)	6 (5.2)
Severe	0 (0)	0 (0)
Treatment-related AEs, n (%)	7 (6.1)	6 (5.2)
Gastrointestinal events		
Abdominal discomfort	5 (4.3)	6 (5.2)
Nausea/vomiting	3 (2.6)	4 (3.5)
Diarrhea	4 (3.5)	5 (4.3)
Oral events		
Oral discomfort	2 (1.7)	1 (0.9)
Oral candidiasis	0 (0)	0 (0)
Dermatologic events		
Rash	1 (0.9)	2 (1.7)
Infections		
Upper respiratory infection	9 (7.8)	11 (9.6)
Acute otitis media	6 (5.2)	9 (7.8)
Serious adverse events (SAEs)	0 (0)	0 (0)
Discontinuation due to AEs	0 (0)	0 (0)
Choking/aspiration events	0 (0)	0 (0)

## 4 Discussion

This study showed that, at the child-level primary endpoint, SSK12 achieved a higher tympanogram classification improvement rate than placebo, with consistent directions across prespecified subgroups; at the ear level, conclusions remained robust after correcting for within-child bilateral correlation using GEE; longitudinal changes in continuous parameters were also consistent, manifesting as increases over time in ΔTPP and ΔYtm and a decrease in ΔTW, with between-group differences gradually widening, forming an effect pattern that corroborated the primary endpoint. Conversion from B/C to A represents restoration of eustachian tube pressure regulation, improvement in tympanic compliance, and normalization of the pressure curve, consistent with the pathophysiological chain of “ventilation improvement—effusion absorption” (Vanneste and Page, 2019; Andresen et al., 2021); after colonization in the oropharynx/nasopharynx, SSK12 suppresses key pathogens through bacteriocin-mediated occupancy and antagonism, reduces local inflammation and mucus viscosity, and improves ciliary clearance, thereby promoting the return of negative middle-ear pressure to normal and clearance of effusion, presenting a time-dependent pathway of microecological remodeling (Tagg et al., 2023; Do et al., 2024); ear-level analyses indicated moderate correlation between the two ears within the same child due to shared nasopharyngeal environment and behavioral exposures, which was adequately controlled by the modeling. Previous randomized controlled studies mostly used acute episodes as endpoints, and evidence on objective middle-ear physiological indicators is relatively scarce (Persaud et al., 2024; Scott et al., 2019). In the context of standardized non-surgical management, this study, with a double-blind design and centrally read tympanogram indices, directly captured improvement in middle-ear ventilation and effusion status, and used ear-level models to verify the lateral consistency and robustness of efficacy, supplementing the evidence base for microecological interventions during the non-surgical management phase of OME, and aligning with the direction of audiological outcomes over the same follow-up period, with clear clinical translational significance. Maintenance of adherence and product viability, successful blinding, and consistency in measurement and reading provided sufficient internal validity support for the above efficacy determinations.

The treatment effect of SSK12 was generally consistent across prespecified age strata and adenoid hypertrophy grades, suggesting broad applicability within the studied population. However, clinical context remains important. Younger children may have greater baseline susceptibility to Eustachian tube dysfunction due to anatomical and immunological immaturity, whereas older children may experience more spontaneous resolution of OME. Similarly, children with moderate adenoid hypertrophy (grade II–III) may derive the greatest benefit from adjunctive non-surgical therapy, as microbial and inflammatory modulation may meaningfully improve Eustachian tube function. In contrast, children with severe adenoid hypertrophy (grade IV), where mechanical obstruction predominates, may be less responsive to conservative measures alone and more likely to require surgical intervention. These considerations support individualized use of probiotic therapy within established clinical decision pathways.

The beneficial effect of nasopharyngeal *Streptococcus salivarius* K12 (SSK12) on middle-ear pressure and tympanogram normalization is likely multifactorial, involving both pathogen suppression and host immunomodulation, rather than a purely antimicrobial mechanism. First, SSK12 colonization reduces the nasopharyngeal burden of key otopathogens, including *Streptococcus pneumoniae*, *Haemophilus influenzae*, and *Moraxella catarrhalis*, through competitive exclusion and production of bacteriocins (salivaricins). Suppression of these pathogens diminishes chronic nasopharyngeal inflammation, which is a major contributor to Eustachian tube dysfunction. Reduced mucosal edema and biofilm burden facilitate restoration of Eustachian tube patency, thereby improving middle-ear ventilation and normalizing tympanometric peak pressure. Second, SSK12 may exert local immunomodulatory effects at the nasopharyngeal mucosal surface. Probiotic colonization has been shown to modulate innate immune responses by downregulating pro-inflammatory cytokines and promoting mucosal immune homeostasis. This immunoregulatory action may reduce persistent low-grade inflammation in the Eustachian tube and middle-ear mucosa, further supporting pressure equalization and effusion resolution. Third, the observed improvement in tympanometric parameters without a proportional increase in systemic antibiotic use suggests a functional recovery mechanism, whereby microbial ecosystem stabilization restores physiological Eustachian tube function rather than merely eradicating infection. The parallel improvements in tympanogram type, DPOAE conversion, and audiometric thresholds support a coherent pathway linking nasopharyngeal microbial balance to middle-ear mechanics. Taken together, these findings support a dual mechanism in which SSK12 acts through (i) suppression of pathogenic nasopharyngeal bacteria and (ii) modulation of mucosal immune responses, leading to improved Eustachian tube function and normalization of middle-ear pressure. Future studies incorporating cytokine profiling and comprehensive microbiome analyses may further elucidate the relative contribution of these pathways.

Microecological and pathogenetic evidence together with clinical endpoints point to a coherent causal chain. SSK12 achieved a high proportion and sustained colonization in the oropharynx/nasopharynx, with load maintained during the intervention period and diverging from the placebo group over time; in parallel, loads of Haemophilus influenzae, Streptococcus pneumoniae, and Moraxella catarrhalis showed continuous downregulation, with significant treatment × time interactions, indicating that a colonization–antagonism effect had occurred at the target anatomical site. The reduction in pathogen load was directionally consistent with decreases in episodes of upper respiratory infection and acute otitis media, while systemic antibiotic prescriptions did not increase, weakening the explanation of apparent improvement driven by rescue medication (Mosquera et al., 2025). Biologically, SSK12, through bacteriocin-mediated inhibition and occupancy competition, attenuates pathogenic adhesion and biofilm formation, together with reductions in local inflammation and improvement in mucus rheology (Stasǩová et al., 2021), thereby enhancing nasopharynx–eustachian tube ventilation, reducing negative middle-ear pressure, and promoting effusion clearance (Hamrang-Yousefi et al., 2020), resulting in time-dependent improvements in tympanogram classification and ΔTPP, ΔYtm, and ΔTW. The reliability of intervention exposure was supported by high adherence and stability of viable counts by batch (Visciglia et al., 2022), and consistency of central reading at the measurement level together with successful blinding reduced the likelihood of information bias and observer bias. Previous studies mostly focused on upper respiratory episodes or subjective symptoms (Merenstein et al., 2024) and lacked objective middle-ear physiological indices synchronized with pathogenetics; in the context of standardized non-surgical management, this trial integrated colonization, load, pathogen spectrum, otologic objective indices, and event outcomes, coherently presenting the pathway “colonization → pathogen decrease → ventilation restoration → classification improvement → hearing benefit,” which more closely approaches causal inference at the mechanistic level than previous evidence and provides methodological and clinical bases for introducing an oropharyngeal lozenge microecological intervention in the “observation period” scenario.

The concurrent assessment of tympanometric, audiological, and microbiological outcomes provides an integrated framework for interpreting treatment effects. Improvements in tympanogram type and middle-ear pressure were accompanied by favorable changes in hearing thresholds and otoacoustic emissions, together with modulation of nasopharyngeal microbial profiles. This convergence supports a biologically plausible pathway in which nasopharyngeal probiotic colonization contributes to improved Eustachian tube function and middle-ear ventilation, rather than reflecting an isolated physiological change. The audiological and otologic functional outcomes were aligned in a beneficial direction with the primary endpoint, with a time-dependent between-group separation in the decrease of the four-frequency pure-tone average and a higher overall probability of distortion product otoacoustic emission (DPOAE) conversion from negative to positive, indicating that restoration of the conductive pathway had translated into perceptible functional benefit; adherence remained high, daily dose exposure was stable, viable counts of the preparation and cold-chain monitoring met standards, and blinding success together with good measurement/reading consistency provided robust internal validity support, while the safety profile was comparable to placebo, rendering the risk–benefit ratio clinically acceptable. Pathophysiologically, reduction of middle-ear effusion and improvement of eustachian tube ventilation can reduce mechanical damping of the tympanic membrane–ossicular chain (Merchant and Neely, 2023; Won et al., 2020), making otoacoustic emissions generated by outer hair cells more readily transmitted through the middle ear to the external auditory canal, so that DPOAE conversion to positive and a decrease in hearing thresholds become functional extensions following improvement in tympanogram parameters (Aithal et al., 2019); after an oropharyngeal lozenge establishes stable colonization in the oropharynx, it maintains the local microecology, together with downregulation of pathogen load and alleviation of inflammation, providing sustained biological impetus for the gradual recovery of the middle-ear conductive chain. Compared with previous intervention studies that used only respiratory episodes or symptoms as endpoints (Paduchova et al., 2024; Darbandi et al., 2021), the present evidence, in the context of standardized non-surgical management, simultaneously presents objective middle-ear physiological indices and audiological outcomes, demonstrating that microecological intervention not only alters the nasopharyngeal ecology but also produces quantifiable auditory improvement within 12 weeks; lozenge administration is convenient for implementation in outpatient and home settings, and adherence data together with quality-control results suggest real-world feasibility of this regimen, strengthening its clinical translational value as a supplementary strategy during the “observation period.”

Economic considerations: The probiotic regimen evaluated in this study represents a low-cost, non-invasive intervention that can be administered in the outpatient setting with minimal resource utilization. In contrast, persistent OME and recurrent AOM are associated with substantial healthcare costs related to repeated clinic visits, antibiotic prescriptions, audiological assessments, and surgical interventions such as tympanostomy tube placement and adenoidectomy.

By improving middle-ear function and hearing during the watchful-waiting period, adjunctive SSK12 therapy may reduce the incidence of AOM episodes, limit antibiotic exposure, and decrease progression to surgery, thereby offering potential cost savings at both the patient and health-system levels. Although a formal cost-effectiveness analysis was not performed, the favorable safety profile, ease of administration, and relatively low acquisition cost of probiotic lozenges suggest that this approach could be economically attractive if clinical benefits are confirmed in larger, multicenter studies.

**Limitations:** This study was conducted at a single tertiary pediatric otolaryngology center, which may limit the generalizability of the findings to other healthcare settings and populations. Differences in patient demographics, microbiological ecology, and clinical practice patterns may influence treatment response. The follow-up duration was insufficient to assess recurrence, long-term hearing and language development outcomes, and it was also difficult to observe differences in surgical outcomes. Event outcomes were infrequent; although the negative binomial model reported aIRR and θ, the statistical power was limited. Information on adherence and events was mainly derived from parental diaries and weekly telephone follow-up, with potential recall and reporting bias. Microecological testing used targeted qPCR and did not characterize community structure, functional genes, or inflammatory profiles, so the causal chain remains incomplete. Eustachian tube dynamics were not directly tested, making it difficult to quantify the physiological magnitude of ventilation improvement. Future multicenter studies across diverse geographic regions and care environments are warranted to confirm the reproducibility and external validity of these findings. Future studies should be multicenter with adequate seasonal coverage and follow-up ≥6–12 months, incorporate objective ventilation indices such as wideband tympanometry and tubomanometry/sonotubometry, and combine 16S/metagenomics and nasopharyngeal inflammatory omics to link the longitudinal mediating pathway among SSK12 colonization, pathogen load, inflammation, and otologic endpoints; use electronic adherence recording and linkage to medical records to reduce bias; design dose–frequency exploration and cost–effectiveness evaluation, and adopt “time to tympanostomy tube insertion” and “long-term hearing thresholds and sustained DPOAE outcomes” as key endpoints to verify and optimize the application strategy of SSK12 during the non-surgical management period. Although the observed associations between nasopharyngeal SSK12 colonization, reductions in otopathogen burden, and improvements in tympanometric and audiological outcomes support a plausible biological mechanism, these microbiota findings are associative rather than causal. As microbial measures were not experimentally manipulated independently of the intervention, causality cannot be fully established from this trial alone. Future studies incorporating targeted microbiome manipulation, longitudinal immune profiling, and mediation analyses will be required to more definitively delineate causal pathways.

## 5 Conclusion

Daily oral SSK12 lozenges for 12 weeks effectively improved tympanogram classification in children aged 3–6 years with adenoid hypertrophy and otitis media with effusion undergoing non-surgical management, manifested as increased probabilities of conversion from types B/C to type A at both the child level and the ear level. Synchronous improvements in continuous tympanogram parameters and audiological function verified the physiological mechanism of restoration of middle-ear ventilation and effusion clearance. Nasopharyngeal microecology analyses showed that SSK12 achieved stable colonization and significantly suppressed common nasopharyngeal pathogens, supporting the causal chain of “SSK12 colonization—decrease in pathogen load—improvement in eustachian tube function—favorable tympanogram classification outcome.” Product viability, successful blinding, measurement consistency, and safety all met quality-control requirements, suggesting that the lozenge can serve as a low-risk, clinically feasible adjunctive intervention during the observation period of non-surgical management.

## Data Availability

The original contributions presented in the study are included in the article/supplementary material. Further inquiries can be directed to the corresponding author.
